# Household ownership and utilization of insecticide-treated nets under the Regional Artemisinin Resistance Initiative in Myanmar

**DOI:** 10.1186/s41182-018-0111-z

**Published:** 2018-07-31

**Authors:** Thae Maung Maung, Jaya Prasad Tripathy, Tin Oo, Swai Mon Oo, Than Naing Soe, Aung Thi, Khin Thet Wai

**Affiliations:** 1 Department of Medical Research, Ministry of Health and Sports, Yangon, Myanmar; 2 International Union Against Tuberculosis and Lung Disease, The Union South East Asia Office, New Delhi, India; 3 International Union Against Tuberculosis and Lung Disease, Paris, France; 4 Population Services International, Yangon, Myanmar; 5 National Malaria Control Program, Ministry of Health and Sports, Nay Pyi Taw, Myanmar

**Keywords:** Malaria, ITN/LLINs, MARC area, Bed net ownership, Migrant

## Abstract

**Background:**

Malaria is a major public health problem in Myanmar with reported artemisinin resistance. Myanmar promotes the use of insecticide-treated nets (ITNs) through the free delivery of long-lasting insecticide nets (LLINs) with target coverage of at least 80% in moderate and high-risk areas by 2014. Migrant people are at greater risk of malaria. They have significant barriers to health care services for febrile illness and malaria. Thus, a community-based survey was conducted among the migrant population to assess the ownership and utilization of bed nets (ITN/LLINs) for malaria.

**Methods:**

The study analyzed secondary data from a community-based malaria survey conducted in 2014 among migrant population in 30 randomly selected townships out of 52 Regional Artemisinin Resistance Initiative (RAI) townships. In each township, five migrant sites were randomly selected (total of 150 migrant sites). A total of 3933 households (approximately 125 households from each township) were selected.

**Results:**

Of 3923 households assessed, 97% had access to at least one bed net (any type), but only half had access to ITN/LLINs. Only 24% of households had adequate ITN/LLIN access (at least one ITN/LLIN per two persons). In terms of household utilization, 94.3% slept under a bed net (any type) the previous night. Only 43.4% slept under an ITN/LLIN. ITN/LLIN utilization in children under 5 years and pregnant women (high-malaria risk groups) was 45.3 and 46.6%, respectively. Of all nets, 31.3% had holes or had already undergone repairs. In terms of insecticide treatment status, 52.9% of bed nets were untreated and 35.9% of ITNs had not been treated with insecticide for more than a year.

**Conclusion:**

This study highlights poor access and high utilization of ITN/LLINs among migrant population, particularly among children and pregnant women. It highlights the need for improving bed net coverage and access to ITN/LLINs through bed net distributions and/or social marketing with the focus on migrant population and targeting of households with children and pregnant women.

## Background

Despite global decline in malaria morbidity and mortality in the last decade, malaria is still one of the major public health problems in Myanmar with two thirds of its population at risk of getting the disease [1]. There is growing evidence of artemisinin resistance in parts of The Greater Mekong Sub-region (GMS), known as the epicenter of multidrug-resistant *Plasmodium falciparum*, especially in eastern Myanmar along with Cambodia and Vietnam. A gradual decline in the therapeutic efficacy of common artemisinin-based combination therapy (ACT) [2] and the evidence of artemisinin resistance in certain pockets of Myanmar [3] led to the Myanmar Artemisinin Resistance Containment (MARC) strategy by World Health Organization (WHO) [4].

The strategy focused on the mobile migrant populations, with a major emphasis on improving access to vector control measures including personal protection, malaria diagnosis, antimalarial drugs, and treatment in order to prevent or delay the spread of artemisinin resistance [4]. In 2014, Regional Artemisinin Initiative (RAI) project was initiated by the Global Fund in 52 selected townships from the MARC areas with suspected artemisinin resistance.

The use of insecticide-treated bed nets (ITNs) is one of the most effective forms of vector control. The WHO Global Malaria Programme has recommended full coverage of long-lasting insecticidal nets (LLINs)/ITNs for malaria prevention [5]. Myanmar also promotes the use of bed nets (ITNs) through the free delivery of LLINs and free treatment of mosquito nets already in use before the start of the peak transmission season. The goal is that at least 80% of people in moderate and high-risk areas are protected by ITNs/LLINs [6]. Previous studies among the general population in Myanmar or among selected migrant groups such as the rubber plantation workers have shown the poor utilization of ITN/LLINs [7, 8].

Migration poses new challenges for health systems. In Myanmar, migrant people are at greater risk of malaria because of their nature of work and proximity to places such as forest, rubber plantation, oil, and mine working places [9]. They temporarily stay in remote areas and have significant barriers to preventive, diagnostic, and treatment services for febrile illness and malaria [7]. Of particular importance is malaria among mobile occupational migrants on international borders, particularly the Myanmar-Thai border where there is a serious threat to ACT effectiveness due to resistance. The 2008 World Health Assembly adopted the resolution on the health of migrants, calling on member states “to promote migrant-sensitive health policies.” Healthy migrants contribute to the positive sustainable development and improve the public health outcomes.

In line with this, a community-based survey was conducted among migrant population in the RAI project areas of Myanmar in 2015. This study aimed to assess the ownership of, access to, and utilization of bed nets (LLIN/ITNs) in RAI areas of Myanmar.

## Methods

### Study design

A cross-sectional study design involving analysis of secondary data from community-based malaria survey conducted among migrant population in 2014.

### Study setting

#### General setting

The Republic of the Union of Myanmar has an estimated population of 51 million inhabitants of whom 70% live in rural areas. The country is bordered by Bangladesh, India, China, Laos and Thailand in the North and East, the Bay of Bengal in the west, and in the south by the Andaman Sea. It is divided administratively, into the capital territory (Nay Pyi Taw Council Territory) and seven states and seven regions. There are 74 districts with 330 townships.

#### Specific setting

Malaria is endemic in 291 out of the 330 townships in Myanmar. The country constitutes of high transmission (> 1 case per 1000 population), low transmission (0–1 case per 1000 population), and malaria-free areas (zero cases) represented by 16, 44, and 40% of the total population, respectively [10]. Malaria remains a public health problem due to climatic and ecological changes, population migration, and ecological development activities such as mining, forestry, and development of multidrug resistance *Plasmodium falciparum* parasite.

Areas are categorized into tiers 1, 2, and 3 according to the evidence and level of artemisinin resistance. Tier 1 areas is where there is credible evidence of artemisinin resistance; tier 2 is where there is a significant inflow of people from tier 1, including those areas immediately bordering tier 1; and tier 3 is where there is no evidence of artemisinin resistance and limited contact with tier 1 areas. There are 31 townships in tier 1, 21 in tier 2, and 258 in tier 3. In 2014, RAI project was initiated by the Global Fund which covered 52 townships from tier 1 and tier 2. The study areas were selected from these RAI project areas (tiers 1 and 2).

Free distribution of ITNs/LLINs in areas of high-malaria transmission is one of the key interventions for malaria elimination in Myanmar. This is done mainly by the National Malaria Control Program (NMCP) and other partners yearly. Although ITN/LLIN is internationally recommended, other bed-net types including cotton, nylon, and lace are available in local markets.

#### Community-based survey among the migrant population

A nation-wide community-based survey was conducted jointly by the Department of Medical Research (DMR) and NMCP in 2014 to understand the knowledge, attitude, and health-seeking behavior towards malaria and the ownership and utilization of ITNs among the migrant population in the selected townships of RAI areas. A total of 3933 households in 125 migrant sites located in 30 townships were covered.

### Data source

Data variables related to the study objectives were obtained from the community-based survey described above. The survey database is available with the Ministry of Health and Sports, Myanmar.

### Study area and population

The survey was carried out in the townships supported by the RAI project which is a Global Fund Grant to avert the spread of artemisinin resistance in the Greater Mekong Sub-region (GMS).

The study population included migrant population residing in the selected townships. However, those who temporarily stay in the deep forest in the selected townships were not enrolled in this study for potential security concerns. The study population (migrant population) was defined as a migrant person who is a male or female at any age who temporarily lives in the selected townships and not registered as a native villager in the village census. Exclusion criteria includes a person who has no intention to stay overnight in the selected area. Household was defined as all persons living under one roof and consuming food cooked from the same kitchen.

### Sampling and sample size

**Fig. 1:**
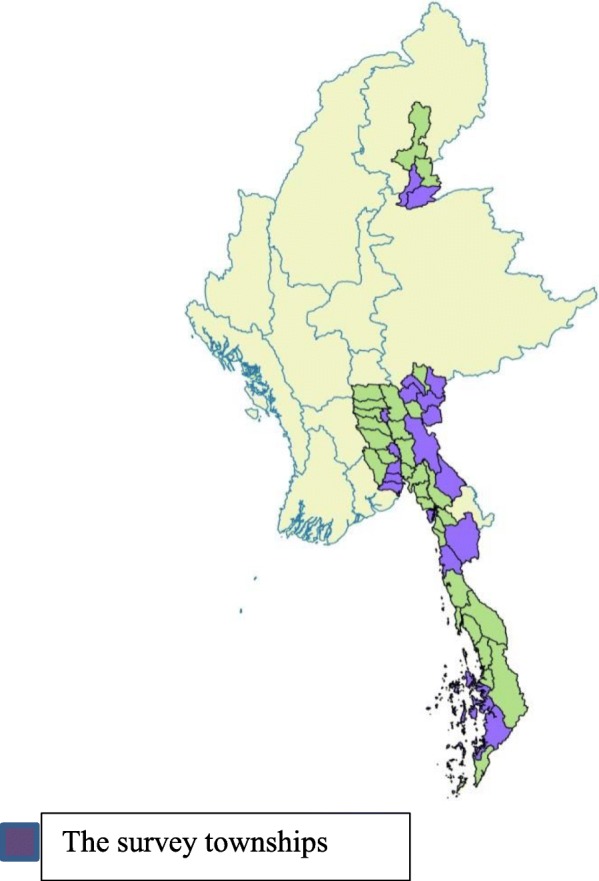
The map showing the survey townships among RAI project townships

Out of 52 townships in RAI areas, 30 were selected randomly. A sampling frame of all migrant sites was available from a migrant mapping exercise done by NMCP staff. In each township, five migrant sites were randomly selected (total of 150 migrant sites). A total of 3933 households (approximately 125 households from each township) were selected. The number of households to be interviewed was based on the probability proportionate to the size of the site. Kawthaung, being a large township, 300 households were selected. A list of households in each site was also available from a baseline census of migrants done prior to the survey. In each site, the households were systematically selected from a list of households (Fig. 1).

### Data validation and statistical analysis

Survey data used for this analysis were double entered and validated using EpiData Entry software (version 3.1, EpiData Association, Odense, Denmark). A descriptive analysis was performed wherein proportions were used to summarize categorical variables related to the ownership and use of bed nets. The ownership of bed nets at household level was assessed using two indicators, (a) % of households with at least one ITN/LLIN and (b) % of households with ITN/LLIN per two household members [1]. Indicators for assessing bed net utilization included the total number and proportion of household members who slept under an ITN/LLIN during the previous night. Information on the physical condition of bed nets was also gathered including material type, presence, or absence of holes and the hole size. Binomial logistic regression was used to determine the factors associated with the ownership of sufficient ITN/LLINs among the migrant population.

The indicators were calculated following the guidelines in the Household Survey Indicators for Malaria Control [11].

1. The proportion of households with at least one ITN/LLIN (number of households surveyed with at least one ITN/LLIN)/(total number of households surveyed)
2. The proportion of households with at least one ITN/LLIN for every two people (number of households with at least one ITN/LLIN for every two people)/(total number of households surveyed)
3. The proportion of population with access to an ITN/LLIN within their household (total number of individuals who could sleep under an ITN/LLIN if each ITN/LLIN in the household is used by two people)/(total number of individuals who spent the previous night in surveyed households)
4. The proportion of population that slept under an ITN/LLIN the previous night (number of individuals who slept under an ITN/LLIN the previous night)/(total number of individuals who spent the previous night in surveyed households)

## Results

### Characteristics of the study population and their household characteristics

Out of a total of 3933 households included in the survey, 3923 (99.8%) households completed the interview. There were a total of 14,331 members in these households. Nearly 52% of them were males and the majority (65%) belonged to the age group 15–59 years (Table 1).

**Table 1:** Demographic characteristics of migrant population in RAI project areas of Myanmar, 2014

Demographic characteristics	Total *n* = 14,331 *n* (%)
Households accessed	3923
Household members	14,331
Gender^a^	
Male	7487 (52.3)
Female	6829 (47.7)
Age group (years)^a^	
< 5	1519 (11.2)
5–14	2755 (20.3)
15–49	7922 (58.4)
50–59	936 (6.9)
≥ 60	431 (3.2)
Household size (*n* = 3923)	
1–2	1185 (30.2)
3–5	2091 (53.3)
≥ 6	647 (16.5)

*RAI* Regional Artemisinin Resistance Initiative

^a^Missing data omitted

About half of the participants lived under a barrack. Nearly three fourths of the population migrated in search of work and nearly two-thirds were unsure of their duration of stay in the present location (Table 2).

**Table 2:** Characteristics and migration dynamics of migrant population in RAI project areas of Myanmar, 2014

Characteristics	Percentage
House ownership	*N* = 3913
Own	1659 (42.4)
Tenant	163 (4.2)
Bar rack	1985 (50.7)
Do not know	3 (0.1)
Others	103 (2.6)
Reason for moving here	*N* = 3917
To work	2940 (75.1)
To find job	780 (19.9)
To live here	122 (3.1)
Visiting relatives/with spouse	239 (6.1)
Other	63 (1.6)
Intention to stay	*N* = 3912
Less than 4 weeks	90 (2.3)
One month to 3 months	231 (5.9)
3 months to 6 months	318 (8.1)
6 months to 1 year	234 (6.0)
More than 1 year	585 (15.0)
Not sure	2454 (62.7)
Next place to move	*N* = 3910
Home	1686 (43.1)
Other work place	401 (10.3)
Do not know	1623 (41.5)
Other	200 (5.1)

### Household ownership, access, and utilization of bed nets

Table 3 shows the household ownership, access, and utilization of bed nets among the migrant population in RAI areas. Almost all (95%) households had at least one bed net per household and, about half (51%) had at least one ITN/LLINs. However, less than one fourth (24%) of all households had sufficient ITN/LLIN, i.e., one ITN/LLINs per two persons.

**Table 3:** Household ownership and utilization of bed nets among migrant population in RAI project areas of Myanmar, 2014

Characteristics	*n* (%)
Household ownership of bed nets	
Total number of households	3923
At least one net per household (any type)	3742 (95.5)
At least one ITN/LLIN per household	1998 (50.9)
One net per two people (any type)	2608 (66.5)
One ITN/LLIN per two people	935 (23.8)
Access and utilization of ITN/LLIN	
Total number of household members^a^	13,592
% of population with access to any bed net	11,558 (85.0)
% of population with access to ITN/LLIN	5723 (42.1)
% of population slept under an ITN/LLIN	4799 (35.3)
% of population slept under an ITN/LLIN in those who have access to LLIN/ITN	83.9%

ITN=Insecticide treated net, LLIN = Long Lasting Insecticide Net

^a^Includes individuals who slept in the household the previous night

Among the household members who slept last night (13592), only 42% had access to ITN/LLINs and only 35% used ITN/LLINs (Table 3). This result shows that high utilization of ITN/LLINs among those who had access to ITN/LLINs.

The proportion of children under 5 years (*n* = 1519) who slept under an ITN/LLIN the previous night was 45.3% while among pregnant women (*n*-178), this was 46.6% (data not tabulated).

### Characteristics of the bed nets including their physical condition

Table 4 shows the characteristics of available bed nets at the household level. Less than half (41%) of all bed nets were ITN/LLINs. The main sources of LLINs were government (63.3%) and NGO’s (32.1%) (table not shown). Around 58% of the bed nets were 2-person size. Of all nets, 31.3% had holes or had already undergone repairs. In terms of insecticide treatment status, an estimated 52.9% were untreated and 38.9% of ITNs had not been treated for more than a year.

**Table 4:** Characteristics of bed nets in households among migrant population in RAI project areas of Myanmar, 2014

Characteristics	Total
Total bed nets	*n* = 6990 *n* (%)
Bed net type	
Non-LLIN	4121 (58.9)
LLIN	2869 (41.0)
Bed net size	6967
One person size	675 (9.7)
One and half person size	352 (5.1)
Two persons size	4037 (57.9)
Family size	1903 (27.3)
Bed net condition	6943
Good (no holes)	4769 (68.7)
Repaired (no holes)	578 (8.3)
Holes	1596 (23.0)
Insecticide treatment status	6922
Untreated	3647 (52.7)
LLIN	2869 (41.5)
ITN	406 (5.9)
Time since last insecticide treatment	387
Less than 6 months	62 (16.0)
6 months to 1 year	186 (48.1)
> 1 year	139 (35.9)

*ITN* insecticide-treated net, *LLIN* long-lasting insecticide net

Missing and do not know categories omitted

### Source of information on ITNs

Regarding the source of information on ITNs, health personnel including private providers were the major informers to the migrant workers (72.4%) followed by leaflets/brochures (8.5%), friends/family members/neighbors (8.2%), and posters (5.6%) (table not shown).

Table 5 shows the factors associated with the ownership of sufficient ITN/LLINs among the migrant population. The associated factors with the ownership of sufficient ITN/LLINs among migrants were state/region, household size, duration of residence since last time, and their intention to stay in the current place.

**Table 5:** Factors associated with ownership of sufficient ITN/LLINs among the migrant population in RAI project areas of Myanmar, 2014

Characteristics	Total	Sufficient ITN/LLIN	Crude OR (95% CI)	*p* value	Adjusted OR (95% CI)	*p* value
	*N*	*n* (%)				
Tier						
Tier 2	1737	302 (17.4)	1		1	
Tier 1	2186	633 (29.0)	1.9 (1.6–2.3)	< 0.001	1.0 (0.6–1.6)	0.996
Education						
Illiterate	346	68 (19.7)	1		1	
Read and write/primary	2900	713 (24.6)	1.3 (1.0–1.8)	0.043	1.1 (0.8–1.5)	0.482
Secondary school	277	52 (18.8)	0.9 (0.6–1.4)	0.782	0.8 (0.5–1.3)	0.443
High school and above	392	100 (25.5)	1.4 (1.0–2.0)	0.059	1.2 (0.8–1.8)	0.372
Occupation						
Daily wage laborer	1775	455 (25.6)	1		1	
Agriculture/animal husbandry	1026	208 (20.3)	0.7 (0.6–0.9)	0.001	0.8 (0.7–1)	0.074
Stone mining work/brick	473	98 (20.7)	0.8 (0.6–1.0)	0.028	1.0 (0.8–1.4)	0.94
Other	627	171 (27.3)	1.1(0.9–1.3)	0.422	1.0 (0.8–1.3)	0.825
State/region						
Tanintharyi	1175	309 (26.3)	1		1	
Kachin	242	18 (7.4)	0.2 (0.1–0.4)	< 0.001	0.2 (0.1–0.3)	**<** 0.001*
Bago	1119	286 (25.6)	0.96 (0.8–1.2)	0.686	0.9 (0.6–1.4)	0.619
Kayah	251	15 (6.0)	0.2 (0.1–0.3)	< 0.001	0.2 (0.1–0.3)	**<** 0.001*
Mon	886	293 (33.1)	1.4 (1.1–1.7)	0.001	1.0 (0.8–1.3)	0.917
Kayin	250	14 (5.6)	0.2 (0.1–0.3)	< 0.001	0.2 (0.02–0.1)	**<** 0.001*
Number of household members						
More than 6	647	71 (11.0)	1		1	
3–5	2091	448 (21.4)	2.2 (1.7–2.9)	< 0.001	2.4 (1.8–3.2)	**<** 0.001*
1–2	1185	416 (35.1)	4.4 (3.3–5.8)	< 0.001	8.6 (6.2–11.6)	**<** 0.001*
Duration of residence since last time						
0–12 months	1872	302 (16.1)	1		1	
13–24 months	580	179 (30.9)	2.3 (1.9–2.9)	< 0.001	2.4 (1.9–3.1)	**<** 0.001*
25–36 months	515	159 (30.9)	2.3 (1.9–2.9)	< 0.001	2.4 (1.9–3.2)	**<** 0.001*
> 36 months	705	251 (35.6)	2.9 (2.4–3.5)	< 0.001	3.1 (2.4–4.1)	**<** 0.001*
Intention to stay						
0–6 months	639	83 (13.0)	1		1	
6 months–1 year	234	45 (19.2)	1.6 (1.1–2.4)	0.022	1.6 (1.0–2.4)	0.044*
> 1 year	585	146 (25.0)	2.2 (1.7–3.0)	< 0.001	1.5 (1.0–2.0)	0.031*
Not sure	2454	657 (26.8)	2.4 (1.9–3.1)	< 0.001	1.5 (1.2–2.0)	0.003*

*represents significant

## Discussion

This is the first community-based study assessing access and utilization of bed nets among different types of migrant population in the Regional Artemisinin Resistance Initiative (RAI) project areas in Myanmar. The present study showed nearly 95% of households had access to bed net of any type, whereas only around half of all households had ITN/LLINs. About 35.3% of households actually slept under an ITN/LLIN last night. Thus, the study reports poor access to ITN/LLIN though utilization is high among those who have access to bed nets.

The major strengths of the study were that data were obtained from a large survey among migrant population in RAI areas, the response rate was high, the interviewers were well trained and supervised, and the subject is an identified national operational research priority. Data quality was ensured through double data entry and validation using EpiData entry software. We also adhered to STROBE guidelines for the reporting of observational studies [12].

The study had some limitations as well. The study did not explore the reasons as to why people do not use bed nets despite possessing them. Further qualitative studies are required to understand the reasons for the same.

The study findings have a number of policy and practice implications. Firstly, only half of the households had access to ITN/LLINs, which is the recommended type of bed net. This is much lower than the desired target of 80%. Migrant population is one of the most vulnerable with high risk for malaria due to their socio-cultural practices and occupation. This suggests the need for urgent attention and community distribution of ITN/LLINs to address the shortage of ITN/LLIN coverage in this vulnerable group. Among the vulnerable mobile population, only about one-third slept under an ITN/LLIN last night which is a matter of concern and warrants urgent attention.

Another large community-based survey in non-MARC areas of Myanmar showed that nearly 58% of households had access to at least one ITN/LLIN, only 24% had one ITN/LLIN per two people and about one-third of people slept under an ITN/LLIN [13]. These figures are similar to those obtained in the present study. This probably points to the fact that apart from the migrant population, there is also poor ownership and utilization of ITN/LLINs in the general population as well.

A study in Kachin region of Myanmar showed high coverage and usage rates of bed nets. The authors ascribed the success to the GFATM rounds for the China Malaria Programme, which distributed free LLINs through Health Poverty Action (HPA) in the region in the past 5 years (2008–2013) ensuring equity through active community participation [8]. Similarly, a report on several African countries showed high-utilization rates in areas with good access [14]. Another study in Myanmar among a small sample of migrant plantation workers reported high coverage, but low utilization of ITNs [7]. Previous studies in various African countries have reported low coverage/ownership and poor utilization of bed nets [15–18]. Few other studies have reported high coverage/ownership but poor utilization of bed nets [19–21]. Matovu et al. also highlighted pro-rich inequalities in ownership of ITNs which were much more pronounced in rural areas [18]. Thus, giving special attention to the poorest and the most vulnerable populations such as the migrants is essential to ensure universal access to ITN/LLINs.

Secondly, it was noted that mass media including television, radio, and newspapers have little role in disseminating information among these migrant workers. This might probably be due to their low-education level and poor socio-economic status leading to lack of access to health education messages through mass media such as radio, TV channels, and press media [7]. This has important implications while designing health promotion campaigns for this vulnerable group.

Thirdly, although the rates of ITN/LLIN use was higher among the children and pregnant women than the general population, only less than half of them (who constitute the high-risk groups for malaria) slept under an ITN/LLIN last night. Since malaria-related morbidity and mortality is highest in these groups [22], more focus and prioritization is mandated to ensure 100% coverage and utilization in these vulnerable subgroups. Studies in different settings have shown poor ownership and use of ITNs/LLINs among pregnant women [23–25] and under-five children [18]. However, in another study in Kachin region of Myanmar, children and pregnant females had higher use rate of ITNs/LLINs [8]. This was probably because of the health behavior and education interventions by the Chinese GFATM Malaria Programme. Under this program, children and pregnant females were prioritized and 390 sessions of community health education were delivered to promote the use of bed nets [8]. This suggests the need for increasing public awareness through community-based activities.

Fourthly, although the study reported poor access to ITNLLIN, there was high utilization of ITN/LLINs among those who had access to it. This shows a positive behavior towards usage of nets, thus pointing to the fact that access to bed-nets is the key barrier to its utilization. The NMCP should direct its resources towards better coverage of ITN/LLIN distribution.

Finally, the majority of available ITNs were either untreated or not treated for more than a year which makes it less effective than treated nets. This pushes the need for LLINs or continuous distribution of LLINs especially for the vulnerable groups, one such group being the migrants in RAI areas.

## Conclusions

The study highlights poor ownership, access to, and utilization of ITN/LLINs among migrant population in RAI areas. But high utilization of ITN/LLINs among those who had access to ITN/LLINs in RAI areas indicates that the need of priority attention should be given to improving the LLIN distribution for migrant populations through effective delivery channels.

## Abbreviations

ACT: Artemisinin-based combination therapy
DMR: Department of Medical Research
GMS: Greater Mekong Sub-region
HPA: Health Poverty Action
ITN: Insecticide-treated nets
LLIN: Long-lasting insecticide nets
MARC: Myanmar Artemisinin Resistance Containment
MOHS: Ministry of Health and Sports
NMCP: National Malaria Control Program
RAI: Regional Artemisinin Resistance Initiative
STROBE: The Strengthening the Reporting of Observational Studies in Epidemiology
WHO: World Health Organization
